# Efficacy and safety of Fufang Xueshuantong Capsules in combination with conventional drugs in the treatment of glaucoma: A meta-analysis and trial sequential analysis

**DOI:** 10.1097/MD.0000000000034202

**Published:** 2023-07-14

**Authors:** Zhicheng Zeng, Pengfei Jiang, Pei Liu, Jun Peng, Qinghua Peng

**Affiliations:** a Department of Ophthalmology, the First People’s Hospital of Guiyang, Chenzhou, China; b Hunan University of Chinese Medicine, Changsha, China; c The First Affiliated Hospital of Hunan University of Chinese Medicine, Changsha, China.

**Keywords:** Fufang Xueshuantong Capsules, glaucoma, meta-analysis, trial sequential analysis

## Abstract

Glaucoma is the leading cause of irreversible blindness worldwide. The aim of this study was to evaluate the efficacy and safety of Fufang Xueshuantong Capsules (FFXST) in combination with conventional drugs in the treatment of glaucoma using meta-analysis and trial sequential analysis (TSA).

**Methods:**

Clinical trials of FFXST for glaucoma were identified in 8 databases until November 2022, and studies were included for meta-analysis and trial sequential analysis.

**Results:**

In terms of efficacy endpoints, meta-analysis showed that the combination group of FFXST significantly improved clinical effective rate (RR 1.29, 95% CI 1.20–1.39, *P* < .00001), visual function (MD 0.04, 95% CI 0.04–0.05, *P* < .00001), light sensitivity (MD 6.07, 95% CI 4.63–7.51, *P* < .00001), end-systolic blood flow velocity (MD 2.68, 95% CI 2.19–3.16, *P* < .00001) and end-diastolic blood flow velocity (MD 2.07, 95% CI 1.86–2.28, *P* < .00001), and significantly reduced total gray-scale value (MD −64.38, 95% CI −69.08 to −59.68, *P* < .00001) and defect of visual field (MD −3.40, 95% CI −4.11 to −2.69, *P* < .00001) compared with the conventional regimen group, while the pulsatility index and resistance index were comparable. The TSA indicated that these benefits were conclusive. In terms of safety endpoints, meta-analysis demonstrated that total drug-related adverse events in the combination group of FFXST were comparable to those in the conventional regimen group, with TSA showing that more studies are needed to validate the current results.

**Conclusion:**

FFXST may be a safety and effective supplementary strategy for the treatment of glaucoma, which is worthy of further research.

## 1. Introduction

Elevated intraocular pressure and insufficient blood supply to the optic nerve may lead to glaucoma, which is characterized by atrophy or depression of the optic nerve, optic nerve damage, and decreased vision, and glaucoma ranks first among irreversible blinding eye diseases.^[1]^ Glaucoma is a group of diseases characterized by optic papillary atrophy or depression, optic nerve damage and visual acuity loss, with elevated intraocular pressure and insufficient blood supply to the optic nerve as risk factors, ranking as the first irreversible blinding eye disease.^[1]^ The global prevalence of glaucoma in people aged 40 to 80 years has been reported to be 3.5%, and with increasing global aging, 111.8 million people are predicted to suffer from glaucoma in 2040.^[2]^ Glaucoma is a serious threat to human health and is the leading cause of irreversible blindness worldwide.^[3]^ The ultimate goal of glaucoma treatment is to protect the optic nerve and retinal ganglion cells, and reducing intraocular pressure and nerve nutrition are commonly used in the treatment of glaucoma.^[4]^ Ophthalmic surgery to reduce intraocular pressure such as laser trabeculoplasty, trabeculectomy, non-penetrating glaucoma surgery and minimally invasive glaucoma surgery, combined with postoperative nutritional optic nerve therapy, can slow the progression of glaucoma.^[5]^ Although drugs that nourish the optic nerve, such as Citicoline, have played a role in improving the prognosis of glaucoma, their clinical efficacy is still limited and the intervention of novel therapeutic strategies is urgently needed.^[6]^ According to reports, Fufang Xueshuantong Capsules (FFXST) has the effect of nourishing the optic nerve,^[7]^ can significantly improve the prognosis of patients after glaucoma surgery,^[8]^ which may have potential clinical value.

FFXST is a proprietary Chinese medicine with most Chinese herbs such as Panax notoginseng, Radix Astragali, Salvia miltiorrhiza and Radix Scrophulariae, which has the function of promoting blood circulation and removing blood stasis, invigorating Qi and nourishing Yin, and has been used clinically for the treatment of stable angina pectoris, cerebral infarction, retinal vein obstruction and glaucoma.^[9]^ In 2012, the first randomized controlled trial of FFXST for glaucoma was published in China, which found that FFXST was effective in slowing optic nerve atrophy in patients with glaucoma.^[7]^ Over the past decade, there has been increasing evidence to support the efficacy of FFXST in delaying the progression of glaucoma and improving patient prognosis as a clinical treatment strategy for consideration. There is no systematic evaluation or meta-analysis of FFXST for glaucoma, and the evidenced-based evidence for the treatment of glaucoma with FFXST remains to be elucidated. Therefore, this study was conducted to investigate the efficacy and safety of FFXST in the treatment of patients with glaucoma, using the conventional regimen as the control group and the combination of FFXST with the conventional regimen as the experimental group.

## 2. Methods

This study strictly followed the systematic review and meta-analysis methodology of the Preferred Reporting Items for Systematic reviews and Meta-Analyses.^[10]^

### 2.1. Literature search

The databases of the China National Knowledge Infrastructure, China Biology Medicine, VIP, Wanfang, Embase, PubMed, the Cochrane Library, and Web of Science were searched for clinical studies of FFXST for the treatment of glaucoma, all with timeframes from build to November 2022. The English subject terms cover “Fufang Xueshuantong” and “glaucoma,” and the Chinese subject terms cover “Fufang Xueshuantong Jiaonang” and “Qingguangyan.” On the basis of the subject terms, the Chinese free words are expanded by China Biology Medicine and the English free words are expanded by MeSH database, and then the subject terms and free words were combined for searching.^[11]^

### 2.2. Inclusion and exclusion criteria

The inclusion criteria are shown below. Type of data: randomized controlled trial. Participants: met the basic diagnosis of glaucoma.^[12]^ Interventions: the control group used conventional regimen such as nerve nutrition, and the experimental group added FFXST on the basis of the control group. Outcomes: clinical effective rate was used as the primary efficacy endpoint, visual function, total gray-scale value, light sensitivity, defect of visual field, end-systolic blood flow rate, end-diastolic blood flow rate, pulsatility index and resistance index were used as secondary efficacy endpoints, and total adverse event was used as the safety endpoint.

The exclusion criteria are listed below. Studies with duplicate publications. Studies with incomplete data. Studies with data that were not available.

### 2.3. Literature screening, data statistics and risk of bias

In the first step, the basic literature retrieved from each database was imported into the Medical Literature King software, and irrelevant literature was eliminated after reading the title, abstract and full text in turn according to the inclusion and exclusion criteria to finalize the included literature. In the second step, the included studies were categorized to extract and record basic information such as authors, year, sample size, sex ratio, mean age, mean duration of illness, interventions, and duration of treatment. In the third step, the risk of bias was assessed using the Cochrane Risk of Bias Assessment Tool according to the required entries. All work was carried out independently by 2 investigators, and any disagreements were adjudicated by a third investigator.

### 2.4. Statistical analysis

Meta-analysis was conducted using Revman5.3, with risk ratio (RR) and 95% confidence interval (CI) as effect sizes for dichotomous variables and mean difference (MD) and 95% CI as effect sizes for continuous variables. Heterogeneity was analyzed by *I*^2^ test and Q test. If *I*^2^ < 50% and *P* > .1, the heterogeneity was small and fixed-effects model analysis was adopted. Otherwise, a random effects model analysis was used. Sensitivity analysis was performed to assess the robustness of the combined results, and the remaining studies were combined for analysis after excluding 1 study at a time. If the results of the sensitivity analysis were not significantly different from the original results, it suggested that the sensitivity of the combination was low and the results were credible. The trial sequential analysis (TSA) 0.9.5.10 Beta software was used for TSA, and if the cumulative Z-value crossed the required information set or TSA bound, the current information was observed to be conclusive.^[13]^ Stata15.0 software was utilized to assess publication bias, and there was no publication bias if the scatter points on both sides of the funnel plot were essentially symmetric and the harbord regression showed *P* > .1.^[14]^

## 3. Results

### 3.1. Literature screening

**Figure 1.**
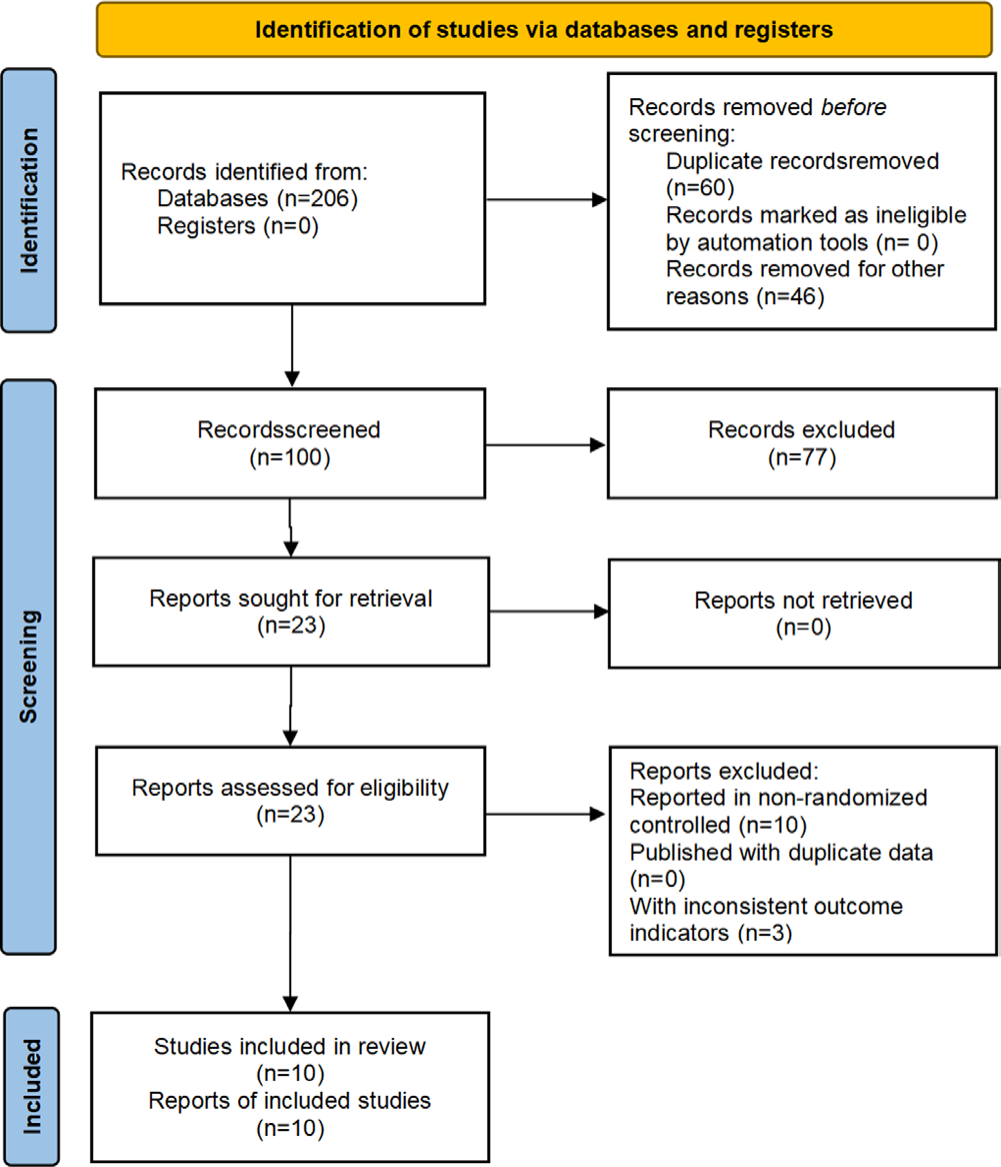
Flow chart of literature screening.

A total of 206 relevant studies were screened, and 106 studies were excluded due to duplication or other reasons. After reading titles and abstracts, 77 studies were excluded. After reading the full text, 13 studies were removed. Ten clinical studies were finally included.^[7,15–23]^ As shown in Figure 1.

### 3.2. Basic characteristics of included studies

A total of 10 clinical studies were included,^[7,15–23]^ with a total sample size of 796 cases, including 399 cases in the experimental group and 397 cases in the control group. All of the study centers in the included studies were located in China. The basic characteristics of the included studies are shown in Table 1.

**Table 1:** Table of basic characteristics of included studies.

Author name	Number randomized	Male (%)	Age (yr)	Disease duration (yr)	Chinese medicine	Conventional therapy	Treatment duration (wk)
Chen JJ 2020	41/41	50.0	55.6	2.4	FFXST 1.5 g tid	Citicoline 0.2–1.0 g qod	40
Li HL 2012	24/23	38.3	/	/	FFXST 1.5 g tid	Nerve Growth Factor 2 mL biw	42
Li J 2018	62/62	58.9	56.0	2.6	FFXST 1.5 g tid	Citicoline 2 mL qod	40
Liu CZ 2015	43/44	44.8	45.5	/	FFXST 1.5 g tid	Mecobalamine 0.5 mg tid	28
Wang Y 2018	54/53	67.3	59.2	1.2	FFXST 1.5 g tid	Vit_B1_ 100 mg qd Vit_B12_ 0.25 mg qd Nerve Growth Factor 30 μg qd	90
Wei QH 2021	34/34	48.5	56.4	/	FFXST 1.5 g tid	Citicoline 0.2–1.0 g qod	40
Yu JP 2019	45/45	54.4	58.3	1.7	FFXST 1.5 g tid	Citicoline 2 mL qod	40
Yuan M 2019	30/30	53.3	65.2	1.8	FFXST 1.5 g tid	Citicoline 2 mL qod	40
Zhang CF 2021	36/35	64.8	56.1	/	FFXST 1.5 g tid	Citicoline 0.2–1.0 g qod	40
Zhang X 2018	30/30	38.3	66.0	1.8	FFXST 1.5 g tid	Vit_B1_ 100 mg qd Vit_B12_ 0.25 mg qd Nerve Growth Factor 2 mL biw	21

FFXST = Fufang Xueshuantong Capsules.

### 3.3. Risk of bias assessment

**Figure 2.**
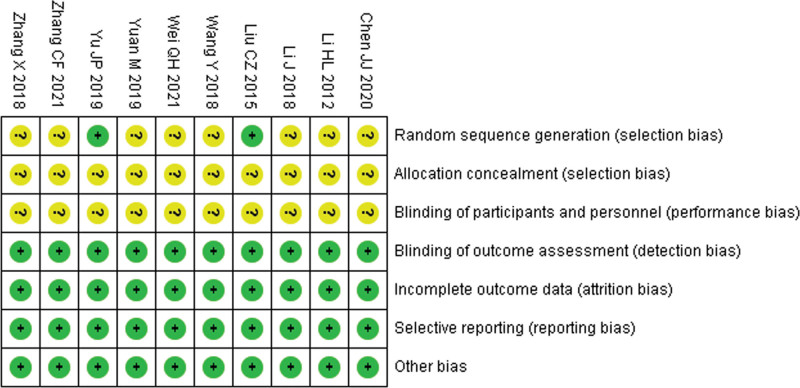
Risk of bias graph.

The risk of randomized methods was unclear in 8 studies, the risk of allocation concealment was unclear in 10 studies, the risk of participant intervention blinding was unclear in 10 studies, and the risk of bias was low in the remaining domains. As shown in Figure 2.

### 3.4. Efficacy endpoints

#### 3.4.1. Clinical effective rate.

**Figure 3.**
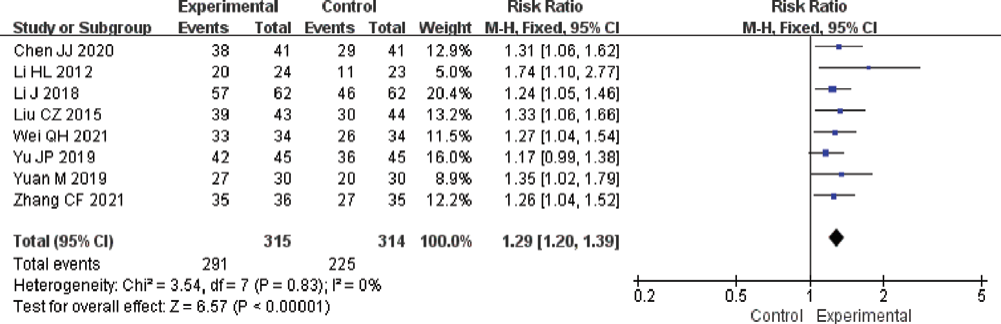
Meta-analysis results of clinical effective rate of FFXST for glaucoma. CI = confidence interval, FFXST = Fufang Xueshuantong Capsules.

Meta-analysis revealed that the combination of FFXST with conventional regimens significantly improved the clinical effective rate (RR 1.29, 95% CI 1.20–1.39, *P* < .00001) in the treatment of glaucoma compared with conventional regimens. TSA demonstrated that the results observed for the current amount of information were conclusive. As shown in Figure 3.

#### 3.4.2. Visual function.

**Figure 4.**
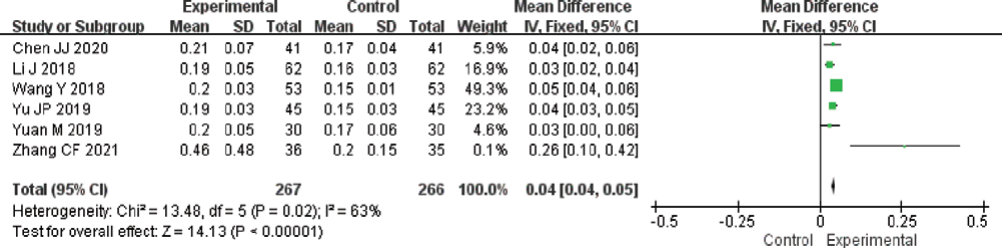
Meta-analysis results of visual function of FFXST for glaucoma. CI = confidence interval, FFXST = Fufang Xueshuantong Capsules.

Meta-analysis indicated that the combination of FFXST with the conventional regimen significantly improved visual function (MD 0.04, 95% CI 0.04–0.05, *P* < .00001) in patients with glaucoma compared to the conventional regimen. Sensitivity analysis showed low sensitivity and high confidence in the results for the combined results of visual function. TSA demonstrated that the results observed for the current amount of information were conclusive. As shown in Figure 4.

#### 3.4.3. Visual field.

**Figure 5.**
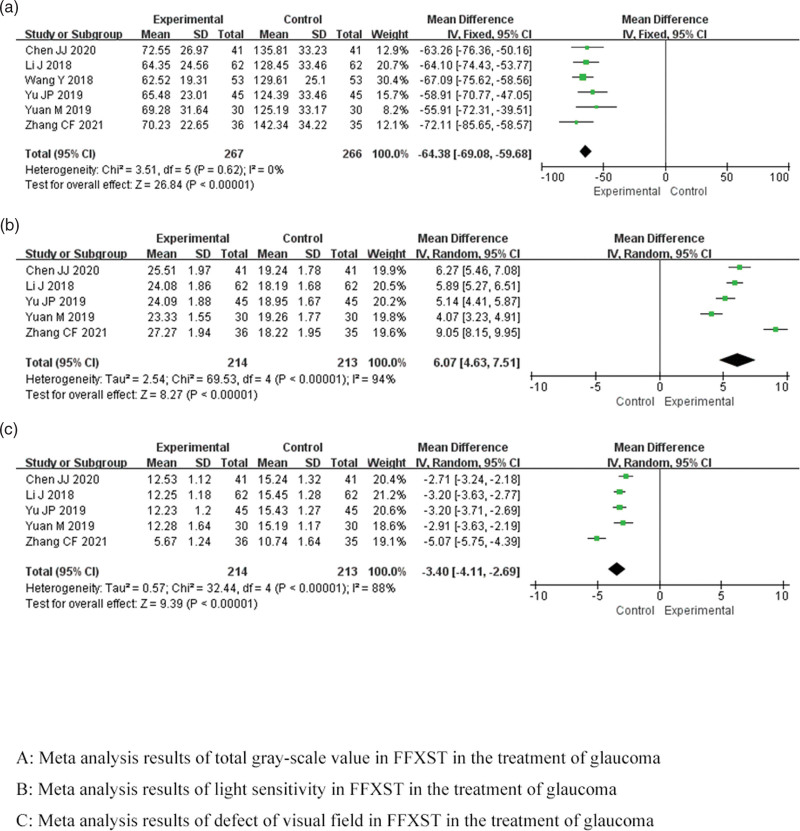
Meta-analysis results of visual field of FFXST for glaucoma. CI = confidence interval, FFXST = Fufang Xueshuantong Capsules.

Meta-analysis indicated that the combination of FFXST with the conventional regimen significantly reduced the total gray-scale value (MD −64.38, 95% CI −69.08 to −59.68, *P* < .00001) and defect of visual field (MD −3.40, 95% CI −4.11 to −2.69, *P* < .00001) and improved light sensitivity (MD 6.07, 95% CI 4.63–7.51, *P* < .00001) in patients with glaucoma compared to the conventional regimen. Sensitivity analysis showed low sensitivity and high confidence in the results for the combined results of defect of visual field and light sensitivity. TSA revealed that the results for total gray-scale value, defect of visual field and light sensitivity were conclusive. As shown in Figure 5.

#### 3.4.4. Ocular hemodynamics.

**Figure 6.**
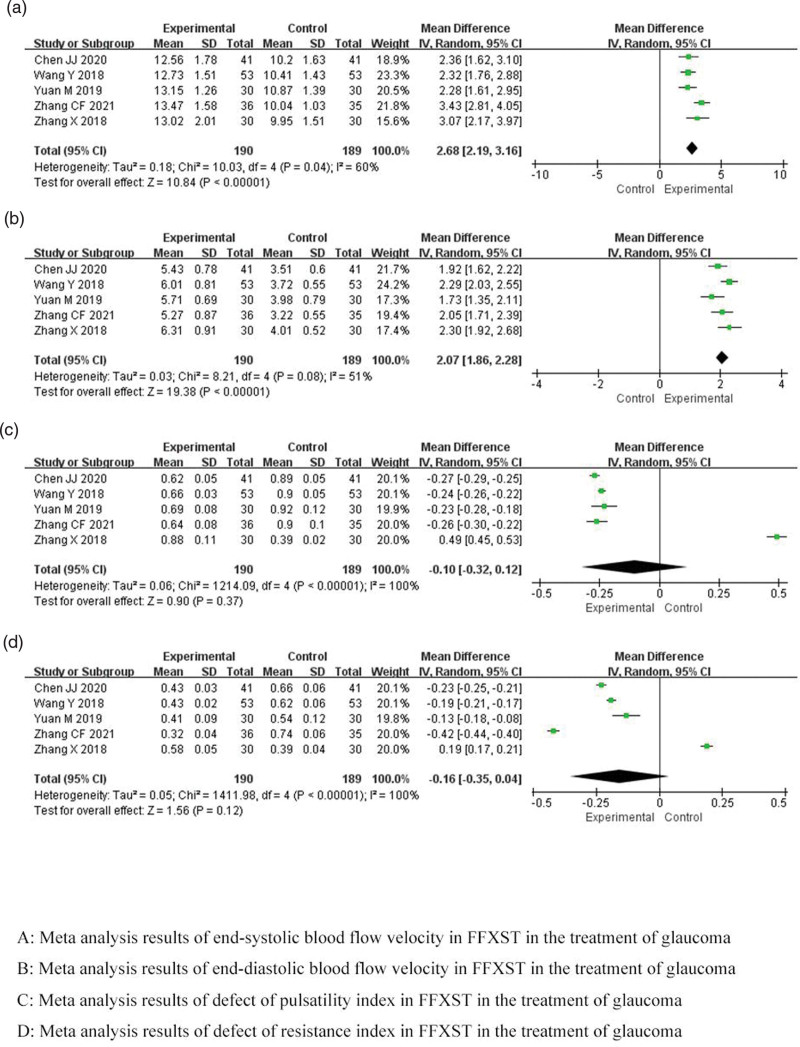
Meta-analysis results of ocular hemodynamics of FFXST for glaucoma. CI = confidence interval, FFXST = Fufang Xueshuantong Capsules.

Meta-analysis demonstrated that the combination of FFXST with the conventional regimen significantly increased the end-systolic blood flow velocity (MD 2.68, 95% CI 2.19–3.16, *P* < .00001) and end-diastolic blood flow velocity (MD 2.07, 95% CI 1.86–2.28, *P* < .00001) in patients with glaucoma compared with the conventional regimen, while the pulsatility index (MD −0.10, 95% CI −0.32 to 0.12, *P* = .37) and resistance index (MD −0.16, 95% CI −0.35 to 0.04, *P* = .12) were comparable. Sensitivity analysis indicated that the combined end-systolic blood flow velocity and end-diastolic blood flow velocity had low sensitivity and high confidence in the results, whereas the combined pulsatility index and resistance index had high sensitivity and low confidence in the results. TSA showed conclusive results for end-systolic blood flow velocity and end-diastolic blood flow velocity, and more relevant studies are needed to validate the results for the pulsatility index and resistance index. As shown in Figure 6.

#### 3.4.5. Safety endpoints.

Safety endpoints were reported in 2 studies. The results showed that the total drug-related adverse events were 0/92 in the combination group of FFXST and 0/92 in the conventional regimen group, and the total drug-related adverse events were comparable. TSA suggested that the current results need to be validated by more relevant studies.

#### 3.4.6. Publication bias.

**Figure 7.**
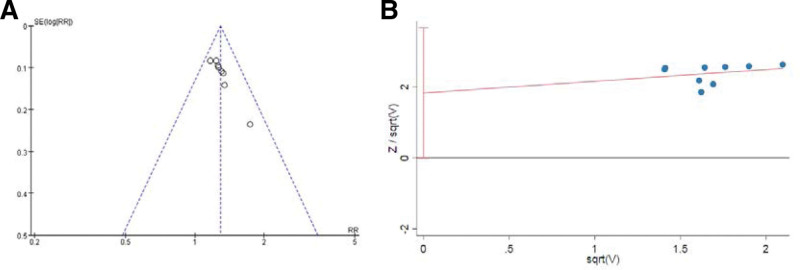
Publication bias assessment.

The inverted funnel plot of clinical effective rate displayed an asymmetric distribution of scatter on both sides, and harbord regression showed *P* = .051, suggesting some publication bias. As shown in Figure 7.

## 4. Discussion

The ultimate goal of glaucoma treatment is to protect the optic nerve and retinal ganglion cells.^[4]^ Although nerve-nourishing drugs such as citicoline, nerve growth factor and mecobalamine have slowed the progression of glaucoma to some extent, their clinical efficacy remains limited.^[5]^ FFXST is a proprietary Chinese medicine with the effect of promoting blood circulation and removing blood stasis, invigorating Qi and nourishing Yin, mainly composed of Panax notoginseng, Radix Astragali, Salvia miltiorrhiza and Radix Scrophulariae.^[24]^ It was initially used in the treatment of coronary artery disease,^[25]^ was first reported to have ophthalmic nerve palsy in 1998,^[26]^ and was first found to improve the prognosis of patients after glaucoma surgery in 2003.^[27]^ As research progresses, there is increasing evidence that FFXST is a potential treatment for glaucoma, but no relevant systematic evaluation or meta-analysis has been published. A total of 10 clinical trials and a sample size of 796 were included in this study, which is the first publication to date to study the efficacy and safety of FFXST in combination with conventional regimen for the treatment of glaucoma.

In terms of efficacy endpoints, meta-analysis demonstrated that the combination group of FFXST was able to significantly improve the clinical effective rate in the treatment of glaucoma compared with the conventional regimen group, and TSA revealed that this benefit was conclusive, suggesting that FFXST were effective in improving the overall efficacy. The combination group of FFXST had significantly higher visual function and light sensitivity and significantly lower total gray-scale value and defect of visual field than the conventional regimen group. TSA showed that these benefits were conclusive, suggesting that FFXST was effective in improving visual function and visual field of patients. In ocular hemodynamics, the combination group of FFXST was able to significantly increase the end-systolic blood flow velocity and end-diastolic blood flow velocity, and TSA indicated that these differences were conclusive, suggesting that FFXST could effectively improve the ocular hemodynamic status of patients. Notably, meta-analysis showed that the pulsatility index and resistance index of the combination group of FFXST were comparable to those of the conventional regimen group. And the sensitivity analysis revealed that after excluding Zhang X 2018, the combined results confirmed that FFXST could significantly reduce the pulsatility index and resistance index. In fact, except for Zhang X 2018, all other included studies believed that FFXST could significantly reduce the pulsatility index and resistance index, with the combined negative results originating from Zhang X 2018. Thus, we considered that FFXST may have the effect of reducing pulsatility index and resistance index, but this result needs to be further demonstrated by more related studies. In terms of safety endpoints, no drug-related adverse events occurred in both the combination group and the conventional regimen group, implying a good safety profile of FFXST.

Although we strictly followed the Preferred Reporting Items for Systematic reviews and Meta-Analyses methodological guidelines for systematic reviews and meta-analysis, this study still had some limitations. First, this study included 10 clinical trials and a sample size of 796, and its research base and total sample size were small, which may reduce the accuracy of the results. Second, the risk of allocation concealment and blinding of participants and personnel for the full study is unclear, which may lead to an increased risk of selective bias and implementation bias. Third, Chen JJ 2020 excluded patients with hepatic and renal insufficiency and Yuan M 2019 excluded patients with combined diabetes or hypertension, and these narrow inclusion criteria may limit the generalizability of the results. Fourth, all of the included studies were conducted in China and the study population was predominantly of Chinese descent, meaning that the findings may only apply to Chinese and it is unclear how beneficial FFXST were in other races. Fifth, because glaucoma requires long-term, regular medication, the long-term effects of the medication are of concern. However, the fact that the duration of treatment in the included studies in this project ranged from 21 to 90 days does not reflect the long-term effects of FFXST on patients with glaucoma. We sincerely expect that future studies will continue to be optimized and explore in depth the clinical value of FFXST in the treatment of glaucoma.

## 5. Conclusions

FFXST combined with conventional drugs can effectively improve clinical effective rate, visual function, light sensitivity, end-systolic blood flow velocity and end-diastolic blood flow velocity, and reduce total gray-scale value and defect of visual field in patients with glaucoma. And with total drug-related adverse events comparable to conventional regimens, FFXST may be a safety and efficacy treatment option for glaucoma.

## Author contributions

**Data curation:** Pengfei Jiang, Pei Liu.

**Methodology:** Jun Peng.

**Software:** Pengfei Jiang.

**Supervision:** Pei Liu.

**Writing – original draft:** Zhicheng Zeng.

**Writing – review & editing:** Qinghua Peng.
